# Self-reported alcohol and drug use six months after brief intervention: do changes in reported use vary by mental-health status?

**DOI:** 10.1186/1940-0640-7-24

**Published:** 2012-10-30

**Authors:** Antoinette Krupski, Jeanne M Sears, Jutta M Joesch, Sharon Estee, Lijian He, Alice Huber, Chris Dunn, Peter Roy-Byrne, Richard Ries

**Affiliations:** 1 Department of Psychiatry and Behavioral Sciences, Center for Healthcare Improvement for Addictions, Mental Illness and Medically Vulnerable Populations (CHAMMP), University of Washington at Harborview Medical Center, Seattle, WA, USA; 2 Department of Health Services, University of Washington, Seattle, WA, USA; 3 Research and Data Analysis Division, Planning, Performance and Accountability Administration, Washington State Department of Social and Health Services, Olympia, WA, USA; 4 Division of Behavioral Health and Recovery, Washington State Department of Social and Health Services, Olympia, WA, USA

**Keywords:** Brief intervention, Comorbid substance use and mental illness, Mental illness, Alcohol use, Binge drinking, Illicit drug use

## Abstract

**Background:**

Although brief intervention (BI) for alcohol and other drug problems has been associated with subsequent decreased levels of self-reported substance use, there is little information in the extant literature as to whether individuals with co-occurring hazardous substance use and mental illness would benefit from BI to the same extent as those without mental illness. This is an important question, as mental illness is estimated to co-occur in 37% of individuals with an alcohol use disorder and in more than 50% of individuals with a drug use disorder. The goal of this study was to explore differences in self-reported alcohol and/or drug use in patients with and without mental illness diagnoses six months after receiving BI in a hospital emergency department (ED).

**Methods:**

This study took advantage of a naturalistic situation where a screening, brief intervention, and referral to treatment (SBIRT) program had been implemented in nine large EDs in the US state of Washington as part of a national SBIRT initiative. A subset of patients who received BI was interviewed six months later about current alcohol and drug use. Linear regression was used to assess whether change in substance use measures differed among patients with a mental illness diagnosis compared with those without. Data were analyzed for both a statewide (n = 828) and single-hospital (n = 536) sample.

**Results:**

No significant differences were found between mentally ill and non-mentally ill subgroups in either sample with regard to self-reported hazardous substance use at six-month follow-up.

**Conclusion:**

These results suggest that BI may not have a differing impact based on the presence of a mental illness diagnosis. Given the high prevalence of mental illness among individuals with alcohol and other drug problems, this finding may have important public health implications.

## Background

Brief interventions (BIs) for alcohol and other drug problems have been consistently associated with decreases in self-reported drinking [1] and, to a lesser extent, decreases in illicit drug use [2,3]. However, alcohol and drug problems do not always occur in isolation. It has been estimated that 37% of individuals with an alcohol use disorder (AUD) and more than 50% of individuals with a drug use disorder (DUD) have a co-occurring mental health disorder [4-8]. Thus, in any screening, brief intervention, and referral to treatment (SBIRT) program, it is likely that a significant subset of persons screened will have mental illness. Because individuals with co-occurring mental illness and substance abuse are known to have poorer outcomes when treated in traditional, non-integrated substance abuse treatment settings relative to individuals without mental illness [9] and often present clinical challenges when faced with traditional interventions based on motivational interviewing (MI) [10], it raises the question of whether such individuals would benefit from traditional SBIRT programs. Yet, few studies have compared the effects of BI on individuals with mental illness and those without.

One such study was conducted in Germany by Grothues and colleagues [11] as part of a larger study of BI with general practice patients identified as having an AUD, at-risk drinking, or binge drinking (N = 408). In an exploratory analysis, reductions in drinking 12 months after BI were compared in a subsample of participants with comorbid depression or anxiety and a subsample of those without. Results indicated that BI was associated with significant reductions in alcohol consumption only in patients without comorbid depression and anxiety. The authors pointed out the comorbid sample was likely too small (n = 88) for differences between groups to be detected.

Although we were unable to identify any other studies that compared outcomes of BI between individuals with and without mental illness, we did identify a number of randomized controlled trials that reported results of comparisons between MI and either treatment as usual (TAU) or another intervention among individuals with co-occurring disorders. Motivational interviewing is typically the basis for BI [12,13], making results of studies employing MI relevant to the present discussion. Although these studies did not include a group without co-occurring disorders, the results provide evidence that MI is associated with decreased alcohol and/or drug use relative to a control condition and suggest that persons with mental illness can benefit from MI. Specifically, two studies used a single brief MI-based intervention similar to the approach used by the SBIRT initiative reported on here. In both, psychiatric inpatients were randomly assigned to either a brief MI session or a control condition, and both reported reduced consumption in the intervention groups [14,15].

We also identified several studies that randomly assigned patients diagnosed with psychosis to intervention and control conditions where the intervention consisted of multiple MI sessions. Results similar to those reported for single-session BI were reported in these three studies [16-18]. (It should be noted, however, that each was based on patient samples of ≤30 people.) Finally, two additional studies explored the feasibility of interventions for alcohol misuse among people with mental illness, the first testing the efficacy of SBIRT among patients presenting to a psychiatric emergency service [19] and the second examining the efficacy of BI conducted by telephone among psychiatric outpatients [20]. Although neither study employed a comparison group, both reported reduced alcohol consumption at six-month follow-up. It is important to note that at least one study found no evidence for an intervention effect in patients with comorbid SUD and mental illness [21].

As noted earlier, many of these studies had methodologic limitations, including low numbers of participants (often due to low engagement rates), unbalanced baseline characteristics across intervention and comparison groups, failure to verify therapist fidelity, and low response rates at follow-up [22]. Nonetheless, taken together, the existing literature provides an encouraging perspective regarding the benefit of drug and alcohol BI for mentally ill individuals. At the same time, results of the one study comparing the impact of BI on individuals with and without mental illness were inconclusive [11]. Thus, the question of whether such individuals benefit from BI to the same extent as individuals without mental illness remains unanswered.

The present study took advantage of an SBIRT program implementation in nine emergency departments (EDs) throughout Washington State as part of a national initiative to establish SBIRT programs in six states, funded in 2003 by the Substance Abuse and Mental Health Services Administration (SAMHSA) [2]. Our goal was to explore differences in self-reported days of alcohol use, days of binge drinking, and/or days of illegal drug use among patients with and without mental illness six months after receiving BI during a hospital ED visit.

## Methods

This was a secondary data analysis of survey and administrative data gathered as part of the Washington State SBIRT (WASBIRT) program. The WASBIRT study protocol was reviewed and approved by the Washington State Institutional Review Board and three institutional review boards that oversee research within specific participating hospitals.

### Setting and intervention

The WASBIRT program began in April 2004, placing chemical dependency professionals (CDPs; i.e., trained substance abuse counselors) in nine hospital EDs throughout the state to screen patients for hazardous alcohol and/or drug use. Patients were screened using two standard instruments: the 10-item Alcohol Use Disorders Identification Test (AUDIT) [23] and the 10-item version of the Drug Abuse Screening Test (DAST-10) [24]. The tests were displayed and scored on a hand-held Personal Digital Assistant (PDA). Immediately upon completion of screening, the CDPs provided a BI lasting 5–10 min to female patients who scored ≥7 on the AUDIT, male patients who scored ≥8 on the AUDIT, and patients of either gender who scored ≥1 on the DAST-10. The AUDIT cutoffs of >7 for women and >8 for men were determined by the WASBIRT protocol [25]. After receiving a BI, patients with an AUDIT score of ≥16 or a DAST score of ≥5 were referred to a counselor for brief treatment [26]. For individuals with evidence of chemical dependency (CD), the counselor was expected to assist them in entering specialized CD treatment.

The BI was based on MI principles that included the use of immediate feedback on substance abuse scores, risks associated with the patient’s pattern of use, advice about self-directed options for change, and reinforcement of personal motivation to change behavior. For training, CDPs underwent a two-day BI workshop to learn the BI tasks mentioned above. Toward the end of this workshop, CDPs participated in a 10-min role-play BI with a trained actor. The role-play was audio-recorded, and the trainer reviewed all recordings and e-mailed written feedback to learners after the workshop. Given the real-world nature of this program, it was not possible to monitor all CDPs’ on-the-job performance on a regular, systematic basis. The trainer did, however, directly observe CDPs on an intermittent basis and provided a booster training session midway through the project. A more detailed description of the setting and intervention can be found in Krupski et al. [27].

### Sample construction

We used two distinct samples for this study: a statewide sample and a Harborview Medical Center (HMC) sample. Working with two samples provided opportunities to replicate results and increase generalizability. As of December 31, 2008, the HMC site had performed approximately 17% of the more than 106,000 screenings conducted by WASBIRT statewide. Located in Seattle, WA, HMC is a comprehensive healthcare facility owned by King County and managed by the University of Washington (UW). It is a primary teaching site for the UW School of Medicine and other UW health sciences programs. The mission of HMC includes serving low-income uninsured patients as well as those with mental-health and substance-use problems [28]. The other eight participating hospitals ranged from a small hospital in a rural area to large hospitals in major metropolitan areas in the state.

As described previously, to be included in either sample, patients had to have received a BI based on an AUDIT or DAST score indicating a potential alcohol or drug use disorder. In addition, patients had to be at least 18 years of age and not in custody. Screening was limited to English-speaking patients at some sites, including HMC, but could include Spanish-speaking patients at sites with the capacity to conduct WASBIRT services in Spanish. Further, patients were included in this study only if they answered three specific questions about alcohol and illegal drug use (described later) at baseline and again at six-month follow-up.

The statewide sample included patients enrolled in either Medicaid (a joint US federal-state program that is the largest source of funding for medical and health-related services for people with limited income) or in a state-funded medical assistance program due to meeting disability incapacity criteria. The HMC sample included patients with mixed payer status: payers included Medicaid, Medicare (a US program that provides health insurance to Americans aged 65 or older, to younger people with disabilities, and to individuals with end-stage renal disease), public or private insurance, and workers’ compensation; however, nearly half of this sample was uninsured. The samples had some overlap; 122 individuals were included in both the state and HMC samples. For the statewide analysis, 828 patients met inclusion criteria, and for the HMC analysis, 536 patients met inclusion criteria.

The PDA used for screening was also programmed to select patients for follow-up using a random-number–generating procedure. Follow-up interviews occurred by telephone approximately six months after BI (range, four to eight months) and were conducted by a highly experienced survey unit from the Washington State Department of Social and Health Services (DSHS). Standard survey techniques were used, including advance contact letters and multiple attempts to call hard-to-reach respondents. Contact information was gathered from patients at baseline and augmented by administrative records if the patient gave permission for this. A toll-free call-in number was provided at baseline and in the contact letters to facilitate contact for those who wanted to be interviewed but might be difficult to reach. In return for participating in the follow-up interview, respondents were sent a check for $20. Approximately 18% of eligible participants were randomly selected for the follow-up survey, and of these, 79% completed it.

### Data sources

The DSHS Research and Data Analysis Division was responsible for collecting and maintaining baseline and follow-up interview responses (including data collected according to reporting requirements of the Government Performance and Results Act Client Outcome Measures for Discretionary Programs), AUDIT and DAST scores, and a record of referrals and interventions provided to participants. Aside from WASBIRT program data, the primary data source for the statewide sample was the DSHS Medicaid Management Information System, which included medical claims and encounter data. The primary data source for the HMC sample was encounter and billing information from HMC medical records. The state’s Treatment and Report Generation Tool (TARGET) is an administrative reporting system of the Washington State Division of Behavioral Health and Recovery that contains encounter-level service records for all individuals who receive publicly funded assessment, detoxification, or CD treatment services. We obtained records of felony and gross misdemeanor arrests in Washington State from the Washington State Patrol.

### Measures

In addition to survey data, we had access to several administrative databases (described in the previous section) that allowed us to control for a large number of potential differences between those with and without a mental illness diagnosis. This information included sociodemographic characteristics, mental health and alcohol/drug-related diagnoses and treatment, arrest history, and health care utilization.

#### Mental illness diagnosis indicator

This indicator (**1:** mental illness diagnosis, **0:** otherwise) was based on the presence of diagnoses of mental illness conditions in billing data for the HMC sample and Medicaid claims or encounter data for the statewide sample for the year prior to the BI. Diagnoses related to drug and alcohol problems were excluded as was the rare diagnosis related to dementia or developmental delay. A concern when using administrative data to identify the presence of a mental illness diagnosis is the accuracy and completeness of diagnostic information. Several analyses provided some reassurance in this respect. First, although unpublished, a recent analysis indicated high agreement for mental illness diagnoses recorded in Medicaid claims and mental health provider records [29]. Similarly, an analysis comparing diagnoses of schizophrenia in Medicaid claims to clinical information obtained from primary mental health providers found that most diagnoses of schizophrenia listed on Medicaid claims are accurate [30]. Finally, an indicator of moderate or high risk alcohol/drug use derived from administrative data was found to serve as an acceptable proxy for self-reported alcohol/drug use obtained from AUDIT /DAST screening scores [31].

In the HMC sample, 59% of participants had no mental illness diagnoses, 31% had depression, 15% had a neurotic/personality disorder, 13% had an adjustment/stress disorder, 12% had mania/were diagnosed as bipolar, and 8% had psychosis. (An individual could have more than one mental-health diagnosis; therefore, the percentages add to more than 100.) This level of diagnostic detail was not available for the statewide sample. We did not attempt to analyze results for diagnostic subsets because of the small numbers of individuals in some of the specific diagnostic categories.

#### Change in self-reported alcohol and illegal drug use

Change in substance use was based on participant answers to the following three interview questions at baseline and at six-month follow-up:

During the past 30 days, how many days have you…

● *used any alcohol?*

● *had five or more drinks in one sitting?* (This was used as a measure of binge drinking.)

● *used any illegal drugs?* (Use of illegal drugs included misuse of prescription drugs.)

Change was calculated by subtracting baseline responses from follow-up responses.

Self-reports of alcohol and drug use in clinical settings have been found to be reliable compared with biological measures [32,33]. A recent study of self-reported substance use in seriously mentally ill patients determined that staff reports mostly agreed with patient self-reports of substance use at an early-intervention clinic for psychosis, where 54.2% of the patients were inpatients and 45.8% of the patients were outpatients [34]. In another study comparing telephone and in-person clinical interviews among patients with bipolar disorder, agreement was good between the two assessments for measures of alcohol use, although patients reported fewer alcohol-related problems during the telephone-administered interviews [35].

*Covariables.* Sociodemographic variables used as covariables, i.e., gender, race/ethnicity, and age, were measured at the BI visit. The approach to measuring covariables that summarized events occurring prior to the baseline visit differed across the two samples due to structural issues with the data. For the HMC sample, the data were organized at the date-of-service level, so that summary covariables were measured over 365 days (one year) or 730 days (two years) prior to the date of the BI (as described below and in Table 1). For the statewide sample, the data were organized by calendar month due to the nature of Medicaid eligibility. For the statewide sample, one year referred to the 12 calendar months (and two years refers to the 24 calendar months) preceding the calendar month that included the BI. Because the calendar month that included the BI also included events occurring after the BI, events occurring in that entire calendar month were excluded from the statewide analysis.

**Table 1:** Sample characteristics

**Variable**	**Statewide Sample**	**HMC Sample**
	N=828 Percent*	N=536 Percent*
Mental health diagnosis in year before BI	43.1	41.2
Gender		
Male	44.9	68.3
Female	55.1	31.7
Race/ethnicity		
Asian or Other	2.9	4.3
Black, Non-Hispanic	13.3	20.3
Hispanic	10.5	6.0
Native American	11.6	6.2
White, Non-Hispanic	61.7	63.3
Age category (in years)		
18-24	27.7	17.9
25-34	25.0	22.0
35-44	24.2	30.2
45-54	18.6	20.9
55 and Over	4.6	9.0
ED visit leading to BI involved an injury	28.9	26.9
Any felony/gross misdemeanor arrest in 2 years before BI	31.4	23.9
Chemical dependency treatment in 2 years before BI	31.3	24.3
Medical/billing records indicated alcohol or drug problem in 2 years before BI	33.6	54.7
AUDIT score category		
Low (score: 0–6 for women; 0–7 for men)	43.5	30.8
Moderate (score: 7–15 for women; 8–15 for men)	29.2	30.0
High (score: 16–19)	7.0	7.7
Very High (score: 20–40)	20.3	31.5
DAST score category		
Low (score: 0)	23.8	24.3
Moderate (score: 1–2)	27.3	28.4
High (score: 3–5)	21.5	19.6
Very High (score: 6–10)	27.4	27.8
WASBIRT sites		
Site 1—Harborview Medical Center	21.9	100
Sites 2 and 3	17.3	N/A
Site 4	15.1	N/A
Site 5	18.2	N/A
Sites 6 and 7	15.6	N/A
Site 8	3.9	N/A
Site 9	8.1	N/A
	Mean (range)	Mean (range)
Number of injuries in 2 years before BI	4.9 (0–99)	0.96 (0–22)
Number of ED visits in 2 years before BI	5.4 (0–207)	2.1 (0–41)
Days from baseline to follow-up survey	167 (145–273)	169 (145–249)
Number of months of Medicaid eligibility in 2 years before BI	16.0 (1–24)	N/A

*Percent at index visit unless otherwise noted.

Arrest history was summarized as the occurrence of any felony/gross misdemeanor arrest in the two years prior to the BI. A variable to control for the number of days between the baseline and follow-up surveys was included, since the amount of lag time might affect the amount of change reported. Alcohol/drug-related variables included (1) an indicator for alcohol/drug-related problems in the two years before the BI (derived using ICD-9 diagnosis codes, procedure codes, and/or sites of service in medical billing/claims data); and (2) an indicator for having obtained CD treatment in the two years before the BI (derived from Washington State’s TARGET database). Health care utilization variables included (1) whether the ED visit at which the BI occurred involved an injury, (2) the number of injuries in the two years before the BI, and (3) the number of ED visits in the two years before the BI.

Because eligibility for Medicaid or the state-funded medical assistance program might have changed over time, the statewide analyses included a continuous variable for the number of months eligible for Medicaid or state-funded medical assistance in the two years before the BI. This was not relevant to the HMC analysis, since billing rather than claims data were used. To control for potential variation between WASBIRT sites, the statewide analysis included indicator variables for seven hospital sites (in two instances, local sites administered by a common health care organization were combined in the analyses). The HMC analyses only included the HMC site.

We categorized AUDIT and DAST scores following the intervention level categories laid out in the AUDIT [23] and DAST [24] manuals, as shown in Table 1. We used indicators for score category rather than continuous score variables in the regression models due to uncertainty that the theoretical effects of BI on change in scores would be linear. For example, a BI might be expected to have more impact for those with high scores compared with either moderate or very high scores.

### Data analysis

We used linear regression to assess whether change in substance use measures differed for those with a mental illness diagnosis compared to those without. All linear regression models included the variables shown in Table 1. Each variable was entered into the models as presented in the table, that is, either as a binary, categorical, or continuous variable. In order to focus on those individuals for whom the BI would be expected to have an impact, specific subsamples of individuals were included in each model. Specifically, for the regression models assessing differences in days of any alcohol use and days of binge alcohol use, the subsample included everyone with an AUDIT score of at least 7 for women and at least 8 for men (so those in the AUDIT-Low category were excluded). For the regression model assessing difference in days of any illegal drug use, the subsample included everyone with a DAST score over 0 (so those in the DAST-Low category were excluded).

Analyses for the statewide sample were performed at Washington State’s DSHS Research and Data Analysis Division using SAS 9.2 for Windows (SAS Institute, Cary, NC). Analyses for the HMC sample were performed at the Center for Healthcare Improvement for Addictions, Mental Illness, and Medically Vulnerable Populations (CHAMMP) at the University of Washington at HMC using Stata/IC 11.1 for Windows (StataCorp LP, College Station, TX). The use of different statistical software was due to differing analyst preferences at the two sites. There was ongoing communication to ensure comparable analyses across sites wherever possible.

## Results

Descriptive information about the two samples (statewide and HMC) is presented in Table 1. At baseline, approximately 49% of the statewide sample reported using illegal drugs in the previous 30 days (15% cocaine, 35% marijuana, 10% methamphetamine, 4% heroin, 6% prescription opiates, and 5% other illegal drugs). Approximately 58% of the HMC sample reported using illegal drugs in the previous 30 days (29% cocaine, 36% marijuana, 9% methamphetamine, 9% heroin, 5% prescription opiates, and 7% other illegal drugs).

Responses to the questions about past-month days of alcohol use, binge drinking, and illegal drug use are presented in Table 2 for each sample. These unadjusted self-reported measures were not significantly different by mental health status for either sample at baseline. Between baseline and six-month follow up, there were notable decreases in the three measures. However, the amount of change was not markedly different by mental health status, except for binge drinking in the HMC sample.

**Table 2:** Baseline, follow-up, and change in self-reported substance use by presence of a mental-health diagnosis

**Measure**	**Mental health diagnosis**	**No mental health diagnosis**	P-value*
	**Mean # days in prior 30**	**Mean # days in prior 30**	
**Any alcohol days**			
Statewide regression sample (n=468)			
Baseline	9.7	9.0	0.48
Follow-up	3.9	4.3	0.60
Change (follow-up minus baseline)	−5.8	−4.8	0.30
HMC regression sample (n=371)			
Baseline	12.0	13.1	0.39
Follow-up	4.8	6.9	0.02
Change (follow-up minus baseline)	−7.2	−6.2	0.40
**Binge alcohol days**			
Statewide regression sample (n=468)			
Baseline	6.6	6.2	0.63
Follow-up	2.0	1.9	0.86
Change (follow-up minus baseline)	−4.7	−4.3	0.73
HMC regression sample (n=371)			
Baseline	9.2	7.9	0.23
Follow-up	2.1	3.3	0.04
Change (follow-up minus baseline)	−7.2	−4.6	0.03
**Illegal drug days**			
Statewide regression sample (n=631)			
Baseline	8.0	7.8	0.87
Follow-up	4.4	4.5	0.95
Change (follow-up minus baseline)	−3.5	−3.3	0.83
HMC regression sample (n=406)			
Baseline	8.8	10.9	0.06
Follow-up	5.1	6.5	0.16
Change (follow-up minus baseline)	−3.7	−4.4	0.56

*P-value for two-sample t-test.

Regression analysis results are presented in Table 3. The amount of difference in change between those with and without a mental illness diagnosis for each of the three measures was an estimated one day or less after controlling for the covariables shown in Table 1. None of these estimates were statistically significant. The only covariables that were consistently significant predictors of change in alcohol/drug use (results not presented) were the AUDIT score categories (significant in the alcohol models but not in the drug models) and the DAST score categories (significant in the drug models but not in the alcohol models).

**Table 3:** Linear regression results comparing those with a mental-health diagnosis to those without

**Model/Sample**		Coefficient*	**95% CI**	**P-value**
Any alcohol days	Statewide (n=468)	−0.01	−2.21, 2.20	.99
Any alcohol days	HMC (n=371)	1.13	−1.46, 3.73	.39
Binge alcohol days	Statewide (n=468)	0.56	−1.44, 2.55	.55
Binge alcohol days	HMC (n=371)	−0.36	−2.74, 2.03	.77
Illegal drug days	Statewide (n=631)	−0.80	−2.70, 1.10	.41
Illegal drug days	HMC (n=406)	0.56	−1.95, 3.07	.66

*The coefficient represents the difference in change from baseline to follow-up for those with a mental health diagnosis compared to those without (a negative coefficient represents a larger relative decrease in use for those with a mental health diagnosis). Each of these models contained all covariables listed in Table 2 (except the HMC sample did not include WASBIRT site variables, since it was comprised of only a single site).

## Discussion

### Summary and implications

Although BI for alcohol and other drug problems has been consistently associated with subsequent decreased levels of self-reported drinking [36] and, to a lesser extent, reductions in illicit drug use [2,3], there is little information in the extant literature as to whether individuals with mental illness benefit from BI to the same extent as those without mental illness. This is an important question because an estimated 37% of individuals with an alcohol use disorder and 50% of individuals with a drug use disorder have a co-occurring mental health disorder [8]; this suggests a significant subset of persons screened in SBIRT programs will have mental illness. The purpose of the present study was to explore the relative effectiveness of BI in individuals with and without mental illness diagnoses and, in so doing, to start the conversation about the effectiveness of BI in individuals with mental illness. Our results indicated no significant differences between those with and without a mental illness diagnosis with regard to the amount of change in hazardous alcohol or illicit drug use over the six-month period between the BI and follow-up, suggesting that BI may not have a differing impact based on the presence of a mental illness diagnosis. As such, they extend the results of an earlier study that compared the impact of BI among individuals with and without a diagnosis of mental illness [11]. They are also consistent with analyses showing that self-reported hazardous alcohol/drug use decreases six months after individuals receive a BI through an SBIRT program [2]. It is relevant to note that the results obtained in our study were based on administration of a standard SBIRT protocol, with no modifications made for individuals who may have been mentally ill at the time of the BI. We appreciate the work of Marino and colleagues [10] and believe that examining the extent to which individuals with diagnoses of mental illness might benefit from a modified SBIRT protocol is an important question deserving of continuing research. We also encourage future studies that include appropriate comparison groups and equivalence testing, as we believe they will help further the conversation about the effectiveness of BI in individuals with mental illness.

### Strengths and limitations

A clear strength of this study was its ecological validity. The intervention took place in real-world hospital EDs throughout the state of Washington, thus increasing the likelihood of generalizability. Another strength was the inclusion of two distinct samples: a statewide sample of those on Medicaid or state-funded medical assistance and a single-hospital, mixed-payer sample that included the uninsured. Findings were comparable for these two samples with only minor exceptions, thus providing evidence for robustness and additional potential for generalizability of the results.

The study’s greatest limitation was the absence of a comparison group. Because of this, the unadjusted changes in alcohol/drug use presented in Table 2 cannot necessarily be attributed to BI. Other explanations for changes in use could include regression to the mean [37], selection factors (e.g., self or CDP selection for screening/BI), behavioral change due to having an injury or due to the ED visit, or the effect of the screening itself. As mentioned earlier, this is an important area for future research.

A second limitation was the use of administrative records to define the presence or absence of mental illness. Although there is some evidence that administrative records can be reliably used as proxies for diagnoses as found in clinical records or surveys, it is not extensive. Thus, there is always the possibility that administrative records may be less accurate and/or complete than clinical records. A third limitation is that we were unable to examine the impact of BI on subgroups based on diagnoses because of the small numbers of individuals in some of the specific diagnostic categories. We agree with Cleary et al. [38] that future exploration of differences between subgroups based on diagnoses would be valuable.

A final limitation relates to the method of analysis. Using our analytic approach, we cannot unequivocally state that the absence of a statistically significant difference in change for those with and without a mental illness diagnosis provided evidence of there being no difference as opposed to there simply being a lack of power to detect such a difference. Moreover, although we believe that an equivalence approach would be a technically superior option for assessing the absence of difference between the groups under study, we deliberately did not employ equivalence testing for this study. The rationale for this decision was that our authors as a group could not agree on an equivalence interval, i.e., the amount of difference that could be considered to have no clinical or public health importance—essential for an equivalence approach. Nor could we identify any substantiated interval in the literature. A reduction (or difference in reduction) in days of use of as few as one or two days could be considered important on a population level and has been considered meaningful in previous state evaluations of BI effectiveness. For this reason, we adopted the analytic approach reported in the paper. By inspecting the confidence intervals provided in Table 3, the reader can judge for themselves what clinical significance the range of likely difference between the two groups may hold.

## Conclusion

Results reported here are consistent with much of the existing literature showing that self-reported hazardous alcohol/drug use decreases six months after individuals receive a BI through an SBIRT Program. They extend existing findings by suggesting that the BI may not have a differing impact based on the presence of a mental illness diagnosis. Given that mental illness is estimated to coexist in 37% of individuals with an alcohol disorder and more than 50% of individuals with a drug disorder, this finding has important public health implications.

## Competing interests

All authors are currently employees of either the University of Washington or the Washington State Department of Social and Health Services and have no conflicts of interest or financial interests.

## Authors’ contributions

AK conceived, wrote, and edited the manuscript and made substantial contributions to analytic design and data/interpretation. JMS designed and executed the analyses and statistical approach, took full responsibility for data management for the HMC sample, and wrote the methods and results sections of the manuscript. JMJ provided methodological and statistical support; she also made substantial contributions to the design of the analysis, interpretation of the data, and review and editing of the manuscript. SE, Director of the Washington State SBIRT Evaluation Project, made substantial contributions to the conception and design of the analysis, acquisition of data, and drafting, review, and editing of the manuscript. LH contributed to the acquisition of data, consulted on the statistical approach, and executed data analyses for the statewide sample. AH made substantial contributions to the design of the analysis and participated in the review and editing of the manuscript. PR-B, CD, and RR participated in the review and editing of the manuscript. All authors read and approved the final manuscript.
